# Finite Element Analysis of Transhumeral and Transtibial Percutaneous Osseointegrated Endoprosthesis Implantation

**DOI:** 10.3389/fresc.2021.744674

**Published:** 2021-11-23

**Authors:** Carolyn E. Taylor, Heath B. Henninger, Kent N. Bachus

**Affiliations:** 1 Department of Orthopaedics, School of Medicine, University of Utah, Salt Lake City, UT, United States; 2 Department of Biomedical Engineering, College of Engineering, University of Utah, Salt Lake City, UT, United States; 3 VA Salt Lake City Health Care System, Salt Lake City, UT, United States

**Keywords:** osseointegration, finite element, endoprosthesis, humerus, tibia

## Abstract

Cadaveric mechanical testing of a percutaneous osseointegration docking system (PODS) for osseointegration (OI) prosthetic limb attachment revealed that translation of the exact system from the humerus to the tibia may not be suitable. The PODS, designed specifically for the humerus achieved 1.4–4.8 times greater mechanical stability in the humerus than in the tibia despite morphology that indicated translational feasibility. To better understand this discrepancy, finite element analyses (FEAs) modeled the implantation of the PODS into the bones. Models from cadaveric humeri (*n* = 3) and tibia (*n* = 3) were constructed from CT scans, and virtual implantation preparation of an array of endoprosthesis sizes that made contact with the endosteal surface but did not penetrate the outer cortex was performed. Final impaction of the endoprosthesis was simulated using a displacement ramp function to press the endoprosthesis model into the bone. Impaction force and maximum first principal (circumferential) stress were recorded to estimate stability and assess fracture risk of the system. We hypothesized that the humerus and tibia would have different optimal PODS sizing criteria that maximized impaction force and minimized first principal stress. The optimal sizing for the humerus corresponded to implantation instructions, whereas for the tibia optimal sizing was three times larger than the guidelines indicated. This FEA examination of impaction force and stress distribution lead us to believe that the same endoprosthesis strategy for the humerus is not suitable for the tibia because of thin medial and lateral cortices that compromise implantation.

## INTRODUCTION

Percutaneous osseointegration endoprosthetic systems are surgically implanted into the medullary canal of amputated bone, and are then passed permanently through the skin and connected to distal exoprostheses. This process bypasses socket suspension, returning limb loading to the bone and proximal joints. Initial introduction was focused on transfemoral limb loss, with increasing utilization for transhumeral and transtibial amputations (1). The Percutaneous Osseointegration Prosthesis (POP) (DJO Surgical, Austin, TX, United States), for example, was developed by the Salt Lake City VA and University of Utah for transfemoral amputees. This system underwent an extensive preclinical evaluation using animal models to refine design characteristics and implantation techniques (2–5). The POP was clinically introduced to 10 patients (Early Feasibility Study, ClinicalTrials.gov NCT02720159) who had improved 1-year post-operative functional outcomes, such as increased bone mineral density and decreased don/doff time (6, 7).

Subsequent transhumeral device development began for percutaneous osseointegration docking systems (PODSs) (8) mimicking the fixation strategy of a transfemoral device with a tapered porous-coated region, circular in cross-section. It is unknown if this same design approach is suitable for the tibia. A morphologic analysis of the tibia suggests that it is possible but may not be appropriate for all residual limb lengths (9).

Uniaxial mechanical testing of the PODS device on a cadaveric tibia assessed initial fixation of the device (Supplementary Materials). In this pilot study, PODS devices were implanted into the tibia and tested for torsion and axial pullout following the methods used for the humerus (8). The results revealed a large discrepancy between initial stability of the PODS system in the tibia compared to the humerus, where failure was 0.21 and 0.7 × that of the humerus in pullout (1,325.1 ± 185.8 N) and torsion (6 ± 2.6 Nm), respectively (8). Before proceeding with the PODS system for transtibial use, we must determine the mechanism for the decrease in initial stability, as this corresponds to the stability of the system early post-operatively when little bone OI has occurred and, worst-case scenario, when no OI occurs. Initial stability also serves as our current predictive measure of long-term stability while we do not have destructive mechanical testing results *in vivo*. Since the tibial morphology indicated a likely fit for the PODS system (9), evaluation of the mechanical interface between the bone and endoprosthesis of the humerus and tibia would determine mechanistically how the same OI region geometry of the endoprosthesis would have a different performance in the two bones. Two primary metrics that correlate to the initial stability of the bone-endoprosthesis interface were examined in this study: (1) impaction force and (2) first principal stress. Impaction force corresponds to the total traction force of the endoprosthesis and initial stability of the system (10). First principal stress corresponds to circumferential stress, which is the primary fracture modality for impaction testing of intramedullary endoprostheses and should be minimized to avoid failure (11–13).

Implantation of PODS devices currently depends on qualitative observation to properly size the residual bone to the endoprosthesis. Implantation instructions indicate that proper sizing is achieved when uniform cortical bone is removed around the distal circumference of the reamer (8). The same instructions were applied to the tibia during mechanical testing (Supplementary Materials). It is possible that the same size selection protocol used successfully for the humerus is not applicable for the tibia, because the medullary canal is less circular and does not have a uniform cortical thickness (9).

Finite element analyses were performed to evaluate percutaneous OI devices for transfemoral use during daily loading (14–17) and failure (18). These studies identified zones of stress shielding that could lead to bone resorption, and stress risers that could lead to bone or endoprosthesis failure. This approach has not been used to evaluate how an endoprosthesis design differs in initial stability for specific anatomic locations.

The goal of this study was to perform finite element analyses (FEAs) to understand the large difference in mechanical failure of PODS between the humerus and tibia. Impaction of the tapered PODS OI region was simulated in the humerus and tibia using cadaver-specific FEA models. Each was implanted with a range of endoprosthesis sizes, encompassing those that contacted the endosteal surface but did not penetrate the periosteal surface, to evaluate the influence of size on impaction force and circumferential stress. We hypothesized that the humerus and tibia would have a different PODS sizing criterion that optimized maximum impaction force and minimum circumferential stress.

## METHODS

### Finite Element Model Description

A total of three humeri and three tibiae were obtained. The use of cadaver tissue was deemed exempted by both the University of Utah Institutional Review Board and the Salt Lake City Veterans Affairs Medical Center (protocol #11755). No demographic information was available for the humeri (one left, two right), but measurements taken from CT scans showed that they were near average in length and cortical thickness (19). The tibiae (two left, one right) were from Caucasian male donors 18, 34, and 46 years old; 172-, 188-, and 183-cm tall; 86, 75, and 91 kg, respectively, and were near average in length and cortical thickness (9). These three representative bones from each anatomic location were selected to understand behaviors of the bone during implantation that correlate to the mechanical results of the same bone to elucidate the mechanism of mechanical failure in the tibia with this endoprosthesis geometry. The bones were scanned using a Siemens SOMATOM Definition Flash (Siemens) scanner (120 kVp, 100 mAs, 512 × 512 acquisition matrix, 1-mm slice thickness) with a bone density calibration phantom (qCT Pro Model 3 CT; Mindways Software Inc., Austin, TX, United States). The bones were then segmented and reconstructed in 3D (MIMICS v23.0; Materialise, Plymouth, MI, United States).

To compare the humerus and tibia more directly, the medullary diameter and average cortical thickness of the humerus at 30% amputation length, where mechanical testing was performed, were best matched to the medullary diameter and average cortical thickness of the tibia, resulting in a 40% amputation length (9, 19). The average diameter of the medullary canal for this distal osteotomy, before any additional bone preparation, was then recorded and corresponded to the indicated size of endoprosthesis according to surgical instructions provided by the manufacturer of the device (20). Each was then virtually reamed to replicate implantation procedures for PODS. A 6-cm tall, 2°-tapered endoprosthesis with a circular cross-section was subtracted from the bone reconstruction to match the prepared inner surface of the bone according to validated procedures for virtual implantation (20). The subtracted reamer was placed at the centroid of the medullary canal and aligned coincidentally to the inertial axis of the medullary canal. The reamer diameter corresponded 1:1 to the endoprosthesis shape when the endoprosthesis was 3 mm proud the distal osteotomy, as measured from the resection to the distal collar of the endoprosthesis. This resulted in ∼0.1-mm radial interference between the bone and endoprosthesis when completely inserted, resulting in dilation of the bone (8, 20).

An endoprosthesis model was created as a 3-cm tall conical geometry with a circular cross section and 2° tapered angle (20). The smaller proximal diameter ranged from 8 to 20 mm, and referenced nominal endoprosthesis size. Each bone model was implanted with many endoprostheses that contacted the endosteal surface but did not penetrate the outer cortex. This allowed for a range of simulated sizes for each bone model to determine the impact of size selection.

**FIGURE 1 |:**
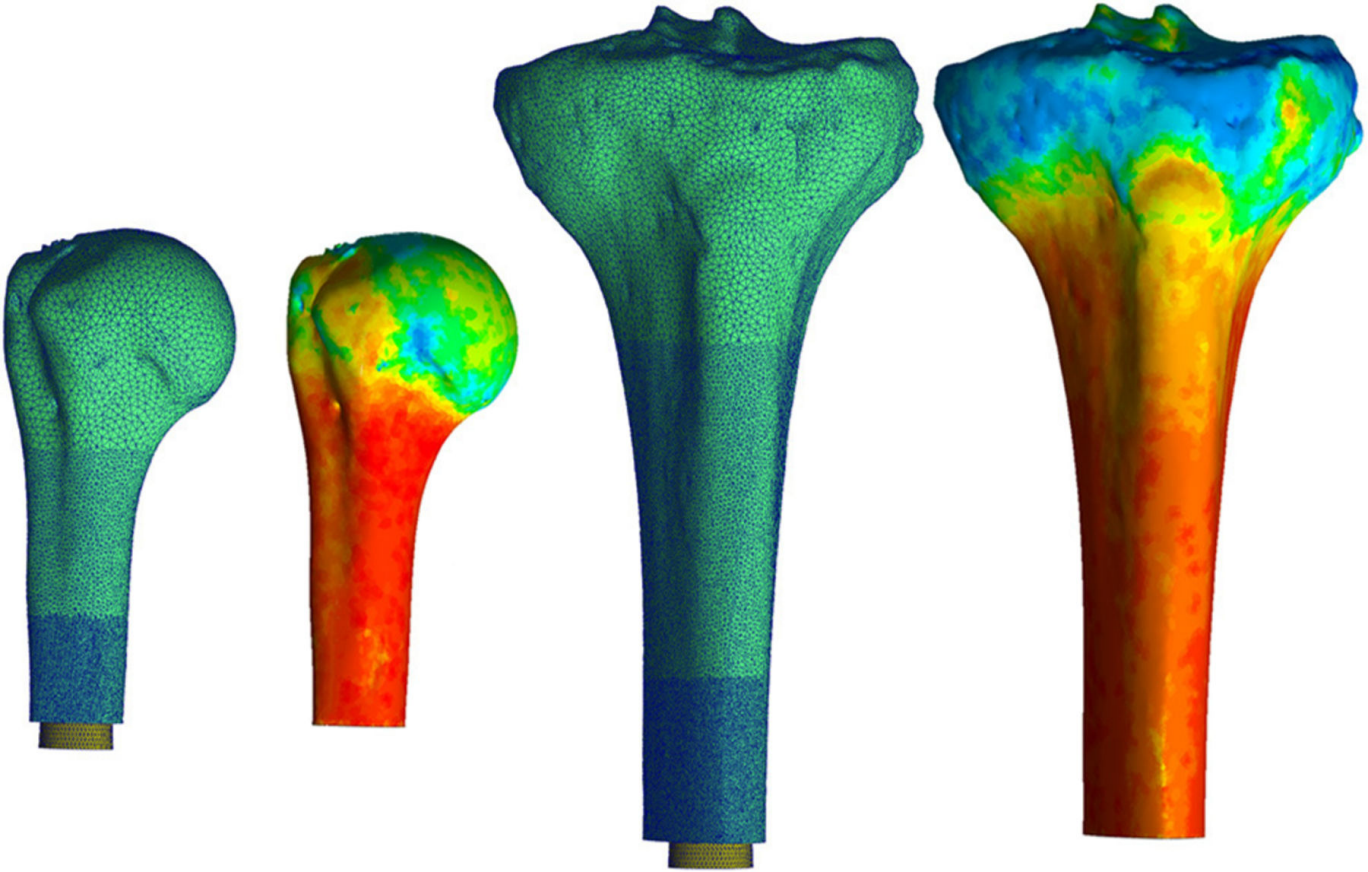
Mesh for both the humerus (left) and tibia (right) began proximally with a coarse mesh controlled with a maximum edge length of 1.2 mm. This progressed into a finer mesh distally with a maximum edge length of 0.2 mm around the endoprosthesis. Heat map represents Young’s Modulus values assigned to each element based on voxel intensity. Pictured humerus and tibia; Young’s Modulus ranged from 0.007 (blue) to 14.8 GPa for the humerus and was 16.8 GPa for the tibia (red). The results of this material assignment were similar for all the other bones modeled. * Relative sizes of the bones are approximately to scale.

Mesh geometry for all the models was constructed using commercially available software, (3-Matic; Materialise, Plymouth, MI, United States) and based on a convergence analysis of one representative tibia, as the region of interest is similar and the modeling approach is the same for both bones. Maximum element edge length dictated mesh parameters because of change in surface area due to different endoprosthesis sizes modeled. Distally, around the endoprosthesis contact zone and through the thickness to the periosteal surface, a maximum edge length of 0.2 mm was used. This grew proximally to a 0.7-mm maximum edge length between the contact zone and the surgical neck of the humerus and tibial tuberosity, followed by a 1.2-mm maximum edge length proximal to these anatomic landmarks (Figure 1). This mesh configuration yielded a 2% difference in impaction force and 5% difference in principal stress compared to a mesh refined to half of those maximum edge lengths. The chosen mesh ran in half the time (22 min, 42 s vs. 48 min 13 s using an Intel Core i5–7600 K processor with 16 GB RAM). The endoprosthesis model had a 0.5-mm maximum edge length across its entirety. All objects were assigned a four-node tetrahedral element. The four-node elements were selected because a 10-node tetrahedral element mesh resulted in only 3% change in force and 0.8% change in stress with 10× the amount of processing time. With small displacements, the use of a rigid body endoprosthesis, and by element-specific property assignment, the four-node tetrahedral elements were determined to be sufficient to represent these data.

Bone density was calculated with linear regression equations derived from the calibration phantom and applied to voxel intensity. These equations were dependent on a CT scanner and settings compared to known density values from the phantom. Young’s modulus of the bone was assigned according to a study that performed regression analysis to correlate tibial, mid-diaphyseal, and cortical bone CT measurements to mechanical and physical properties (21):

$$E=0.06^{*}\rho^{0.74}$$

All voxels ≥ 100 HU were equally divided into 10 uniform subgroups based on a convergence analysis; an increase to 20 subgroups resulted in only 3% decrease in maximum principal stress and 1% decrease in final impaction force. The median value for each subgroup became the assigned Young’s modulus and density for each element in the subgroup (Figure 1). A lower-bound Young’s modulus of 0.007 GPa and density of 0.05 g/cm^3^ were assigned to all voxels ≤ 100 HU. No assumptions were made on the overall distribution of the material properties of the bone, since material property assignments of each element were made based on the voxel intensity of the CT scan compared to the calibration phantom in the field of view.

All amputated bone and endoprosthesis meshes were imported into FEBio Studio (v1.0, FEBio Software Suite, febio.org) (22). The bones were assigned neo-Hookean material properties but maintained the element-specific Young’s modulus and density values and a uniform Poisson’s ratio of 0.3 (17). It should be noted that FEBio automatically converts Young’s modulus and Poisson’s ratio to Lamé parameters, since this model is not solved as linear elastic and strains did not exceed infinitesimal strain assumptions. Since the endoprosthesis is made of titanium, which is much denser and stronger than bone, the endoprosthesis was assigned a rigid body material to simplify the FEA.

**FIGURE 2 |:**
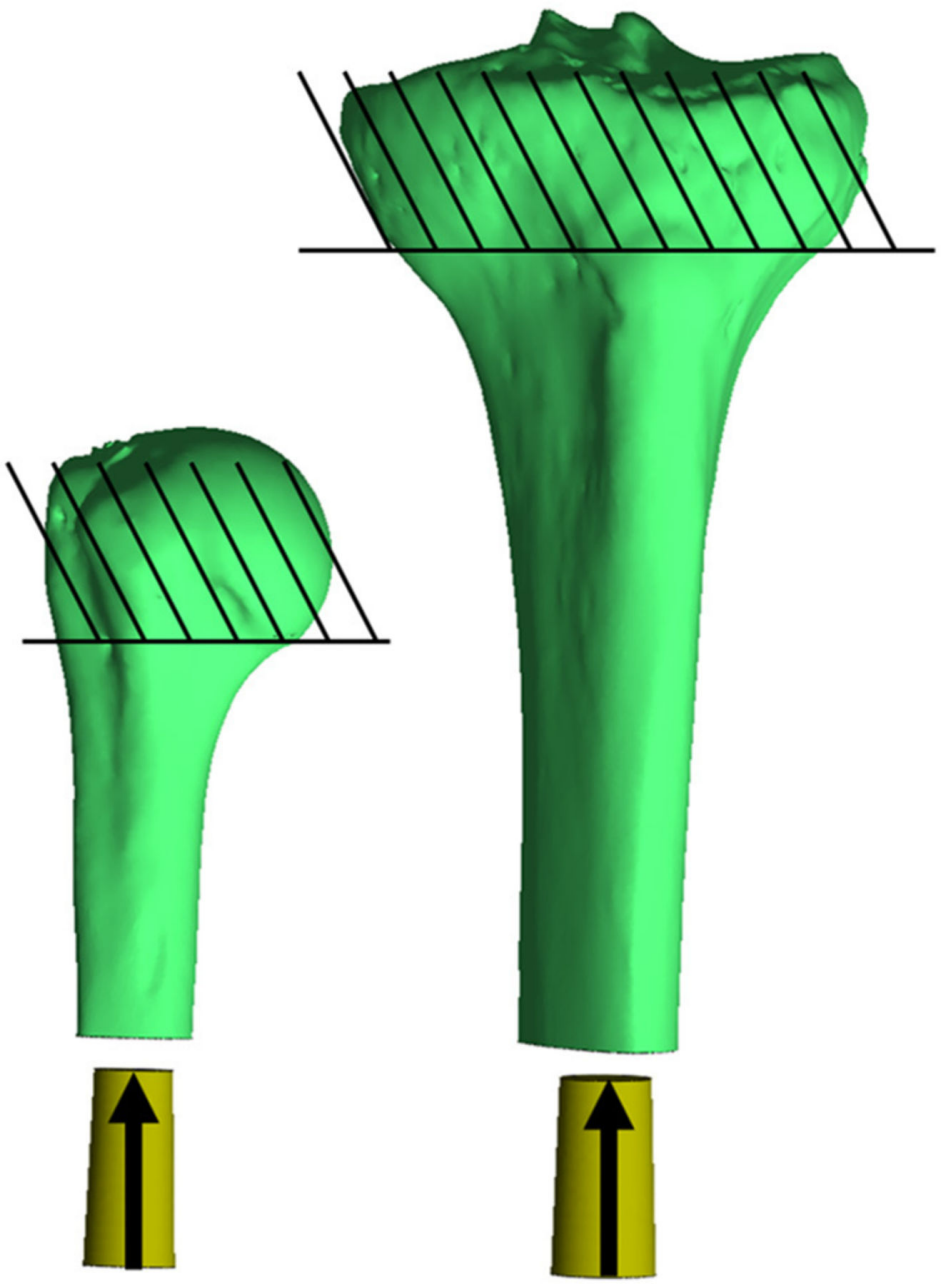
Impaction of the endoprosthesis along the long axis of the bone through the centroid of the medullary canal, parallel to the inertial axis of the medullary canal. The endoprosthesis was placed at the distal end 5mm proud the distal osteotomy, so no contact occurred with the bone (30mm to the bone from where it is currently pictured). Contact began with the endoprosthesis 3mm proud the distal osteotomy, and subsequent impaction created a maximum of ∼−0.1mm mismatch in diameter of the prepared bone endosteal surface to the endoprosthesis. Bones were fixed proximal to the humeral head and tibial tuberosity. *Relative sizes of the bones are approximately to scale.

Final implantation was modeled with a displacement ramp function simulating a quasi-static press-in condition over 1 s. The bone was fixed in all directions proximal to the surgical neck of the humerus and tibial tuberosity. The endoprosthesis began 5 mm outside the distal osteotomy (no contact with the bone) and was moved into place so contact began after 2 mm displacement and terminated when the distal end of the endoprosthesis was flushed to the distal osteotomy. The endoprosthesis was fixed in all degrees of freedom except along the long axis of the bone, which was aligned with the implantation axis of the endoprosthesis, and the endosteal surface of the medullary canal was prepared (Figure 2). A sliding elastic contact was assigned between the two objects with a coefficient of friction of 1.3 determined by testing the PODS porous coating (P^2^ Porous Coating, DJO Surgical) on cancellous bone foam (23).

### Cadaveric Testing

Physical impaction tests were completed on the three cadaver tibias to quantify the force of experimental impaction. Each bone was prepared according to the device manufacturer (8). Bone preparation stopped when the endoprosthesis could be placed in the medullary canal 3 mm proud the distal osteotomy. A part comparison analysis (conducted in 3-Matic) of CT reconstructions was performed to verify the accuracy of virtual implantation used for FEA models compared to prepared cadaver bones. The RMS error between the two surfaces was recorded.

Finally, constructs were loaded onto a material test machine (Model 858 Mini Bionix II; MTS Systems, Eden Prairie, MN, United States) with a 25-kN load cell (#622.2OH-05; MTS Systems, Eden Prairie, MN, United States) so that loading was along the long axis of the bone and endoprosthesis. By displacement control, the machine pressed the endoprosthesis in place at a rate of 5 mm/min, terminating at a set displacement measured with calipers between the distal osteotomy and endoprosthesis end loading collar. The speed was selected based on an initial evaluation of sampling rate on the force vs. time curve to ensure that accurate force was captured without extensive interpolation among points. Force and displacement data were acquired at 1 kHz.

### Data Analysis

The final maximum rigid force of the endoprosthesis in response to the bone, corresponding to the impaction force, was recorded over the impaction period for FEA models. Additionally, circumferential stress, corresponding to the circumferential stress around the bone, was recorded. Sizes of all the endoprosthesis models were compared to the average diameter of the medullary canal at the distal osteotomy, and rounded to the nearest whole number for consistent comparison independent of medullary canal diameter and nominal endoprosthesis size.

The amount of bone-endoprosthesis contact at final implantation was also recorded as a percentage of the possible contact surface area of the endoprosthesis (20).

## RESULTS

### Finite Element Models

**FIGURE 3 |:**
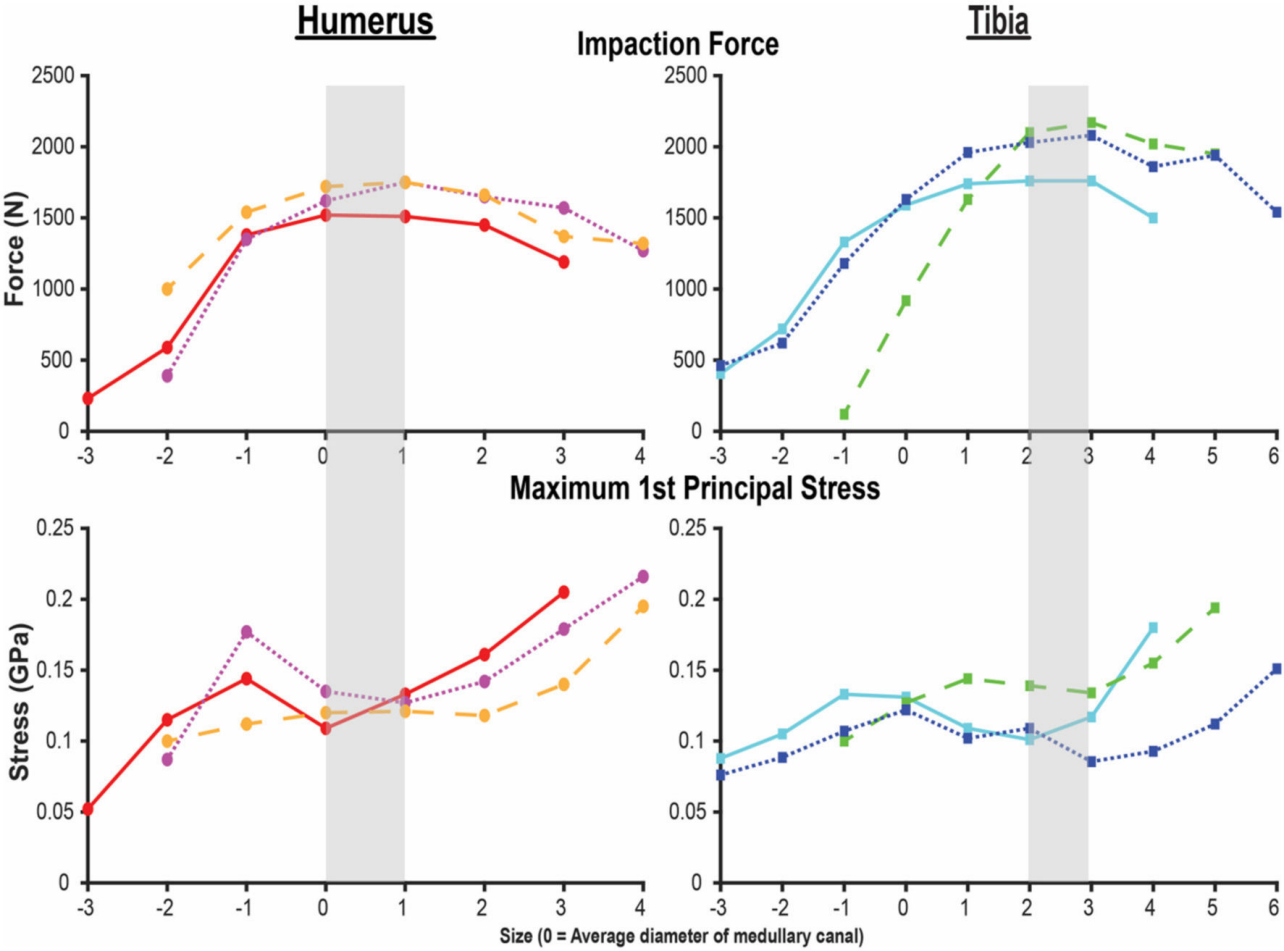
Final impaction force (top) and maximum first principal stress (bottom) for the humerus (left) and tibia (right) finite element analysis (FEA) models. X-axis is the endoprosthesis size normalized to the average diameter of the medullary canal. Each line represents one of the three humeri and tibiae modeled by FEAs. Shading indicates sizing that maximizes impaction force while minimizing stress. Small peaks and valleys in the overall pattern occurred in areas of a subject-specific feature of the medullary canal.

A total of 21 humerus models were constructed from the three bones with indicated endoprosthesis sizes of 10, 10, and 11 mm. These included endoprostheses barely contacting the endoprosthesis around the distal osteotomy and increasing until just before there was penetration through the periosteal cortex at the distal osteotomy. A total of 25 tibia models were created from the three bones with indicated sizes of 11, 14, and 15 mm and the same size range criteria. All cases were normalized to the average diameter for that particular bone (size 0), resulting in a size comparison from a minimum of −3 to maximum of +6 (Figure 3). Each step corresponds to a radial increase of 1 mm in the diameter of the endoprosthesis. The humerus and tibia models had an average of 349,451 and 542,684 elements, respectively.

Both the humerus and tibia models followed similar trends in impaction force and circumferential stress (Figure 3). Impaction force had a sharp increase to a maximum at a normalized endoprosthesis size of +0–1 for the humerus and +3 for the tibia. The force then decreased slightly as the endoprosthesis size continued to increase. The circumferential stress increased, plateaued, or decreased slightly, and then increased again for all the models (Figure 3). The plateau occurred at +0–2 for the humerus and +2–3 for the tibia before increasing again as endoprosthesis size increased (Figure 3).

**FIGURE 4 |:**
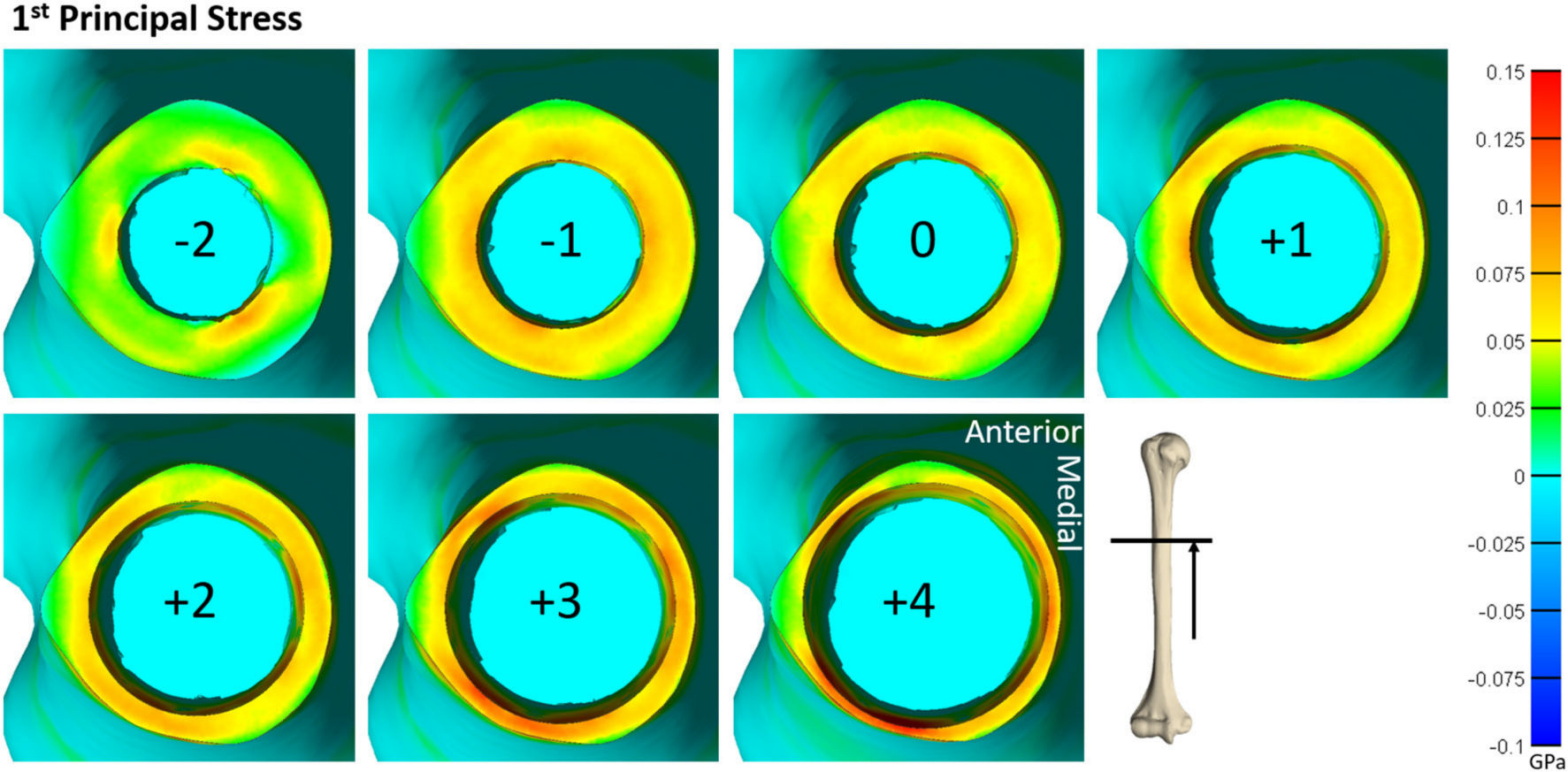
Heat map of the first principal stress (circumferential stress) for each size of implanted endoprosthesis for one of the humerus models. For small endoprosthesis sizes, maximum stress occurred around subject-specific morphologic features. Once the endoprosthesis made contact with more areas of the bone (around size 0), a more uniform stress distribution was observed. Peak stress also began propagating through the thickness of the bone at the distal end around size +3.

**FIGURE 5 |:**
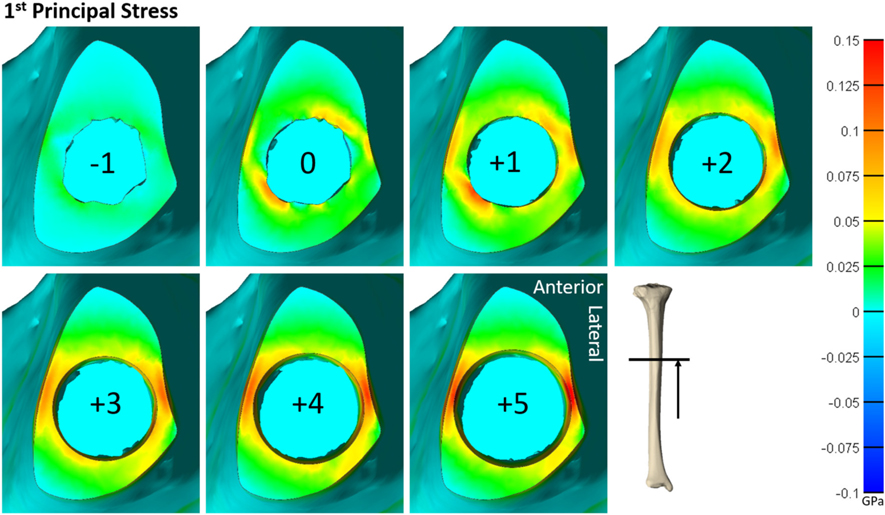
Heat map of the first principal stress (circumferential stress) for each size of implanted endoprosthesis for one of the tibia models. For small endoprosthesis sizes, maximum stress occurred around subject-specific morphologic features. Once the endoprosthesis made contact with more areas of the bone (around size 3), peak stress was uniformly distributed around the medial and lateral regions, and began to propagate through the thickness of the bone at the distal end.

Qualitative observations of the stress field revealed that the maximum stress was concentrated in thin-walled regions. This was more dispersed for the humerus (Figure 4) but concentrated in the medial and lateral regions for the tibia (Figure 5). Subject-specific morphologic features created smaller stress concentrations around the medullary canal, especially for smaller-size endoprostheses where these features were not removed by reaming (Figures 4, 5). This was more pronounced in the tibia where the medullary canal was more elliptical than in the humerus, meaning a larger endoprosthesis was necessary before making contact around the circumference and removing more model-specific morphologic features.

Resultant contact area at final implantation revealed that humeral implantations achieved 58.4 ± 11.2% contact at the indicated size implant, while the tibia achieved 40.2 ± 26.1% bone-endoprosthesis contact (Table 1). The humerus achieved more than 13% bone-endoprosthesis contact for sizes −1–2 compared to the tibia.

### Cadaveric Testing

A part comparison analysis revealed an average RMS error between surfaces (range) of 0.24 mm (0.15–0.33 mm). Testing revealed that the FE models overestimated impaction force by 334 ± 124 N (mean ± STD) (Table 2).

## DISCUSSION

Our primary objective was to determine the mechanism that causes a large discrepancy in mechanical failure data of the same endoprosthesis for the humerus and tibia. We sought to evaluate the influence of endoprosthesis size on impaction force and circumferential stress in both anatomic locations. We hypothesized that the humerus and tibia would require a different sizing criterion in order to use the same PODS design to maximize impaction force and minimize circumferential stress. This hypothesis was confirmed, as the optimal sizing for the humerus corresponded to implantation instructions, whereas the optimal sizing for the tibia was three sizes larger than the instructions indicated. These results are specific to the press-fit, tapered porous-coated region with a circular cross-section of the PODS system and may not necessarily translate to other fixation approaches currently in use for percutaneous OI attachment systems, such as threaded screws (24). The results also varied with longer systems that apply a press fit to a bigger region of the bone (25).

During mechanical tests of PODS devices on the humerus and tibia of humans, fractures were observed along the long axis of the bone, primarily in thin-walled regions. Similar fracture patterns in the femur during impaction of total hip replacements (11–13) suggests failure due to circumferential stress arising from the dilation of the bone from endoprosthesis interference. The circumferential stress was analyzed and showed an intermediate plateau region with 12 ± 11% and 12 ± 5% stress variance in the humerus and tibia (Figure 3), respectively. This plateau occurred around sizes +0–2 or −1 to 1 in the humerus and +1–3 or +1–4 in the tibia. Failure stresses in radial dilation of cortical bone have not been well characterized. However, the most comparable study on ultimate stress performed compressive failure testing on bone plugs taken from the radial and circumferential axes of the femur, and found an ultimate stress of 0.063 and 0.065GPa, respectively (26). These results do not provide a direct measure of stress from radial dilation of the bone, since both sides are fixed, but they provide a fracture risk threshold in the correct loading direction of observed fractures. Other tests that have characterized transverse properties of cortical bone report an ultimate stress of 0.131 GPa (27). Almost every case examined by FEA had a maximum circumferential stress within this range (Figure 3). However, below a normalized size of +3, this was a localized point around a specific morphologic feature of the medullary canal and did not propagate through to the outer cortex (Figures 4, 5). Stress propagation to the outer cortex would increase the risk of periprosthetic fracture instead of localized fracture to a small intramedullary feature. Targeting sizes before heightened stress propagation and in the plateau of lower stress is ideal to minimize stress while maintaining high impaction force.

Higher impaction force correlates to tighter fit and increased initial stability (10) of the bone-endoprosthesis interface as long as it does not create too much stress and increase risk for fracture. In the humerus, maximum impaction force occurred in sizes +0–1, meaning the average diameter of the medullary canal at the distal osteotomy is a good indicator of endoprosthesis size that maximizes impaction force. In the tibia, this occurred in size +3. There is a large difference between these two bones in decrease in impaction force after the maximum. For the humerus, the difference in impaction force from size +1 to +2 was only 83 ± 21 N (4.9 ± 0.9%). In the tibia, the difference in impaction force from size +3 to +4 was 210 ± 56 N (10.8 ± 3.9%). This indicates that there is little room for error when trying to achieve maximum impaction force while not fracturing the bone, especially in the tibia at this examined amputation level.

In the humerus, the stress plateau coincided with peak impaction force (Figure 3). In the tibia, peak impaction force was at the high end of the plateau just before the sharp increase in stress (Figure 3). As a result, there is more room for error to achieve the maximum impaction force with a smaller stress for the humerus compared to the tibia, providing a possible explanation as to why the mechanical testing results (Supplementary Materials) (8) were so different between the bones, beyond the fact that optimal sizing is different. In mechanical testing of the tibia, endoprostheses were three sizes smaller than the optimal size the FEA predicted, decreasing impaction force and associated stability.

**FIGURE 6 |:**
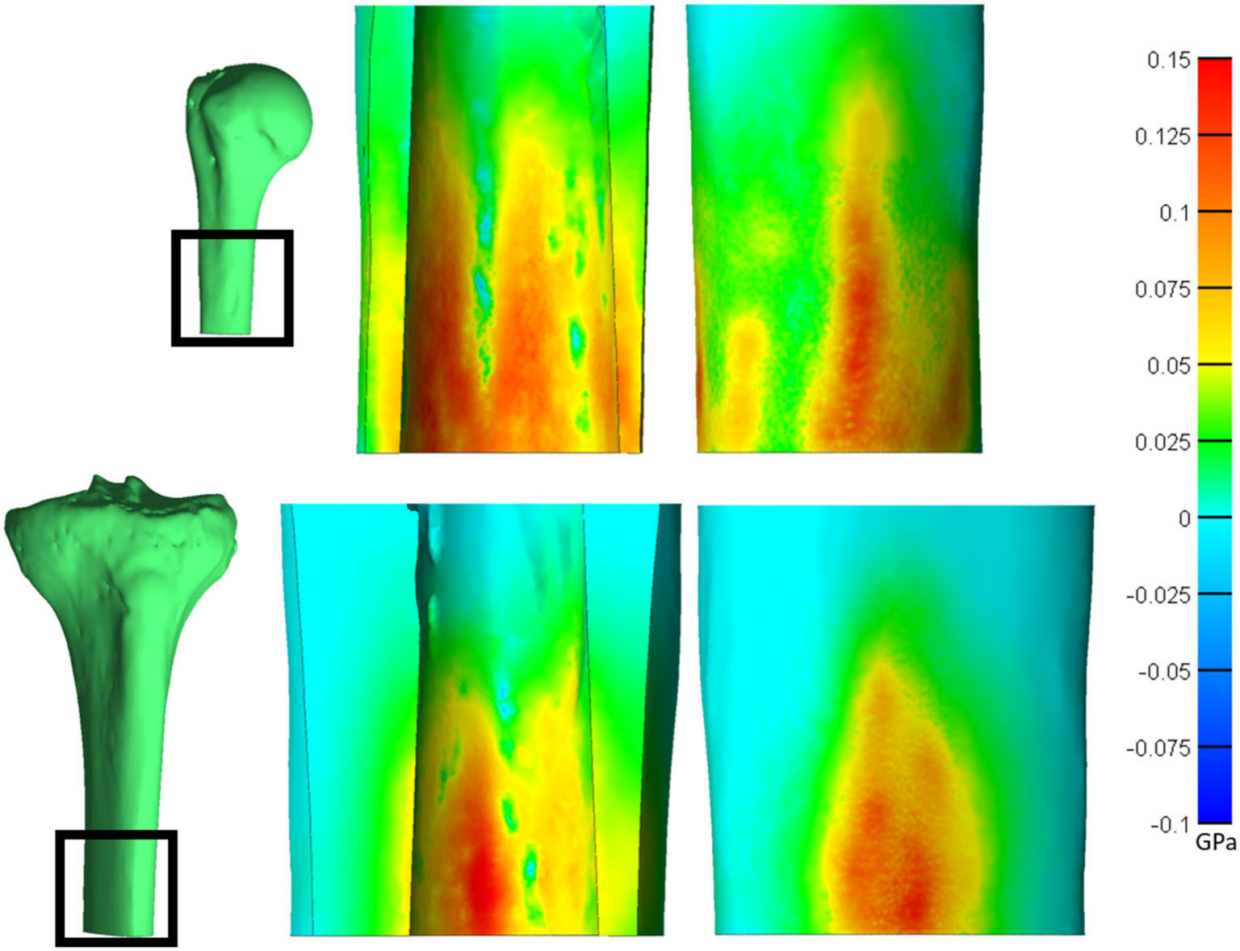
Saggital cross-section of the humerus (top) and tibia (bottom) showing the endosteal (middle) and periosteal (right) surfaces. Heat map represents the first principal stress. Both bones have a normalized size of +3. *Relative sizes of the bones are approximately to scale.

**FIGURE 7 |:**
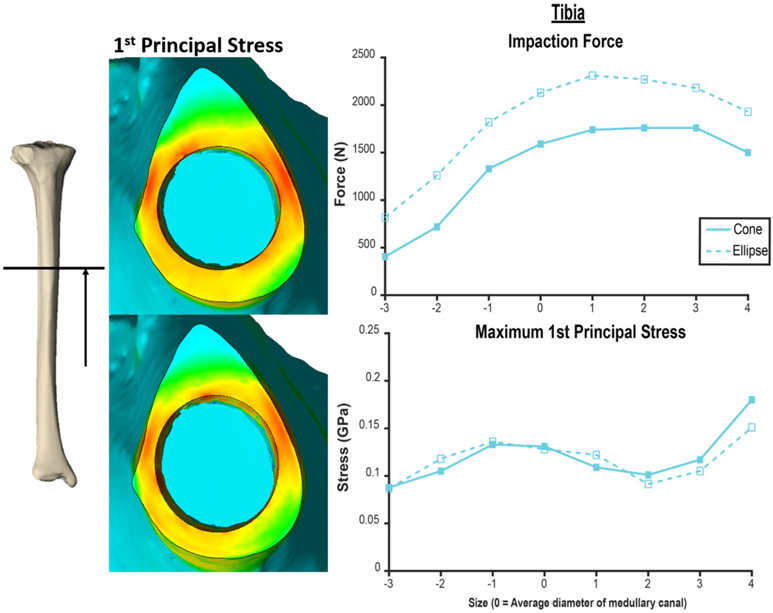
Comparison of tapered endoprosthesis with circular cross-section (left top, right solid) and endoprosthesis with tapered elliptical cross-section (left bottom, right dashed). The elliptical endoprosthesis has a 1-mm larger radius of major and minor diameters. The minor diameter of the ellipse matches the diameter of the circular endoprosthesis. This comparison was conducted on the same tibia bone model. Heat map scale of first principal stress (left) is uniform across both models.

The humerus experienced a more uniform stress distribution around the circumference of the endoprosthesis because of the uniformity of cortical thickness (Figure 6). The tibia had very thin-walled medial and lateral sides and concentrated stress to specific regions with larger endoprosthesis sizes (Figure 6). This localized high stress in the tibia highlights a pattern that would benefit from a different design approach that preserves the medial and lateral cortices to maintain cortical thickness during impaction, like an elliptical cross-section (28). To test this concept, we modeled a tapered, elliptical endoprosthesis with a major diameter 2 mm larger than the diameter of the circular cross-section geometry, aligned to the anteroposterior axis, and held all other parameters constant. We then implanted it into one of the modeled tibias and included the same array of endoprosthesis sizes, with revised preparation so that interference would remain the same for the new shape (Figure 7). This pilot test maintained circumferential stress but showed a 489 ± 62 N increase in impaction force for all endoprosthesis sizes (Figure 7). This increased initial stability is due, in part, to the achievement of more contact with the bone and preservation of thin cortex regions creating more resistance to deformation and more uniform distribution of the stress around the endosteal surface. Further research should determine how aggressive this ellipse should be to accommodate the morphology of the tibia. Based on the current results, we hypothesize that increasing the major diameter of the endoprosthesis would increase impaction force without significant change in stress until the cortex in the anterior and posterior regions are thinned similar to the medial and lateral regions. Further analysis is required to confirm this hypothesis. Clinically, the surgical instrumentation necessary to achieve this elliptical shape would be more difficult to design. We propose using the same reamer geometry followed by a broach with increasing major diameter to achieve this cross-sectional shape.

Other cross-sectional geometries may also perform better than the circular cross-section, and the highly variable morphology of the tibia along the long axis may benefit from several amputation-level specific design approaches. However, this increases implantation cost with multiple tooling sets. This problem needs to be addressed and balanced to optimize mechanical stability while allowing for implantation in a wide population of individuals with transtibial amputation.

Validation of the model is necessary to confirm the observed stress field and make more specific claims about how this endoprosthesis design performs. This validation should include strain gauge measurements or optical tracking of strain to validate beyond impaction force to inform the model. The cadaveric testing showed that the current model parameters were of good approximation but overestimated the impaction force by 333.8 ± 123.6 N (Table 2). This could have been influenced by the use of four-node tetrahedral elements that are a more rigid element type and the fact that the coefficient of friction used is determined by testing cancellous bone foam due to the unavailability of a value determined on cortical bone. Besides tuning model parameters, there were factors in the cadaveric testing that could cause the disagreement. The models also did not simulate the porous coating on the endoprosthesis that files away bone when impacted. Also, the actual endoprostheses have a diametric variation of the porous coating, meaning the size modeled in the FEA might not exactly match that used in cadaver tests. Additionally, experimentally measuring 3 mm proud the distal osteotomy with calipers adds error in the preparation, because interference between the endoprosthesis and bone is not perfect. This could increase forces if the endoprosthesis was more than 3 mm proud and decrease them if less.

This study is limited in that the models were constructed based on a small sample size of non-amputee bones. Heterotopic ossification, osteoporosis, cortical thinning due to disuse atrophy, and other changes in bone morphology are common for lower extremity amputees (29–31), and would decrease the impaction force and stress in patient populations with lower bone quality. The full range of endoprostheses that did not penetrate the periosteal surface was examined to try to capture the case of very thin cortex possible for amputees with disuse atrophy of the bone. A small, representative sample size was selected to begin to elucidate the mechanisms causing observed mechanical failure. We did not employ statistical shape modeling for bone geometry, because we also performed mechanical testing on the same cadaver bone and used the CT scan of the cadaver to determine element-specific mechanical properties. This correlation between mechanical testing results and FEA models would not be possible with a statistical shape model. Once a revised endoprosthesis has been designed that improves on previous initial stability results, FEAs using statistical shape models would be beneficial to assess the new design on a wider population.

Furthermore, only one amputation level for both the humerus and tibia was modeled. Investigation of more amputation levels would determine if these findings indicate a new design in the tibia is applicable to the entire length of the bone. At amputation levels more distal to 40%, a circular cross-section may be suitable, since there is a more uniform circular medullary canal and cortex (9), but long amputations may not present as good candidates for percutaneous OI attachment because of prosthetic component height (20). Additional studies are necessary to refine FEA models and investigate device designs before implementing a percutaneous OI endoprosthesis into the population of transtibial amputees.

This study modeled the impaction of the PODS porous-coated OI region in the humerus and tibia by FEA. Forces and circumferential stresses were recorded for impaction with an array of endoprosthesis sizes, revealing that current implantation protocols are optimized for transhumeral implantation but not for tibial implantation. The tibia requires an endoprosthesis with a diameter larger than previously predicted for the same PODS OI region in order to achieve maximum impaction force, but this quickly causes an increase in periprosthetic stress. In order to achieve safe implantation of a transtibial endoprosthesis, we recommend further investigation on an endoprosthesis with elliptical cross-section based on the preliminary investigation of this device and failure to achieve acceptable results with the current PODS system.

## Supplementary Material

Supplementary Materials

## Figures and Tables

**TABLE 1 |:** Bone-endoprosthesis contact (%).

Normalized size	Bone-endoprosthesis contact (%)					
	Tibia 1	Tibia 2	Tibia 3	Humerus 1	Humerus 2	Humerus 3

−4		5.4				
−3	7.9	13.4		6.8		
−2	20.7	22.9		21.8	17.6	3.7
−1	37.5	34.7	1.2	41.4	44.9	27.0
0	58.9	51.3	10.4	64.7	65.1	45.6
1	76.7	70.7	27.0	83.6	83.9	65.1
2	93.7	89.5	49.4	100.0	99.7	84.2
3	100.0	100.0	70.8	100.0	100.0	100.0
4	100.0	100.0	91.2		100.0	100.0
5		100.0	100.0			
6		100.0				

Amount of bone in contact with the endoprosthesis as a percentage of the totalpossible contact surface of the endoprosthesis.

**TABLE 2 |:** Results of impaction force from FEA and cadaveric tests on the same tibia bone.

Tibia specimen	Average Young’s Modulus (GPa)	Final impaction force (N)	
		FEA	Cadaver

1	11.02	2,101	1,741
2	10.04	1,958	1,758
3	9.45	1,326	884

Average Young’s Modulus was calculated from calibrated CT scans of cadaver bones used in mechanical testing and informing material properties ofelements in the bone mesh for FEA.

## Data Availability

The raw data supporting the conclusions of this article will be made available by the authors, without undue reservation.
