# Supplementary material for: Finite Element Analysis of Transhumeral and Transtibial Percutaneous Osseointegrated Endoprosthesis Implantation
Source: Front Rehabil Sci. 2021 Nov 23;2:744674. doi: 10.3389/fresc.2021.744674 (PMC8849523; doi:10.3389/fresc.2021.744674)
Supplement: Supplementary file 1 [file Data_Sheet_1.docx]

**Supplementary Materials: Mechanical failure testing of the tibia**

**Methods:**

Fresh-frozen tibia specimens were cleaned of all soft tissue and amputated at 40% residual length. Residual length was measured from the tibial plateau to the tibial plafond. Each specimen was surgically prepared for Percutaneous Osseointegrated Docking System (PODS) endoprosthetic implantation using a straight reamer, planar, and broach. The reamer dictated endoprosthetic size. This included subsequent reaming with an initial size of 9 mm and proceded until uniform bone removal was achieved at the distal osteotomy. Planar and broach tooling finished preparation. The broach was used until the endoprosthesis could be placed in the medullary canal 3 mm proud as measured between the end loading collar of the endoprosthesis and the distal osteotomy. Endoprostheses were then impacted with a surgical hammer until fully seated in the medullary canal with contact of the distal end loading collar.

Specimens were then loaded on a servo-hydraulic material test machine (MTS 858 Mini Bionix II, MTS Systems, Eden Prairie, MN, USA) with a 25 kN and 250 Nm load cell (#622.2OH-05, MTS Systems). Specimens were randomly assigned to undergo either axial pullout or torsional testing. Axial pullout tests were performed with a 10 N tensile preload at a rate of 5 mm/min until failure and torsional tests were performed with a 0.5 Nm preload a rate of 1 degree/sec. Force, torque, and displacement were recorded at 1 kHz.

**Results:**

Peak force and torque were recorded for axial pullout and torsional testing, respectively.

| **Specimen #** | **Sex** | **Age** | **Side** | **Size** | **Test** | **Fracture** | | **Ultimate Failure (N)** |
| --- | --- | --- | --- | --- | --- | --- | --- | --- |
| 1 | M | 28 | R | 14 | Axial Pullout |  | | 206.0 |
| 2 | F | 15 | L | 15 | Axial Pullout |  | | 282.3 |
| 3 | M | 18 | L | 15 | Axial Pullout | X | | 440.5 |
| 4 | F | 21 | R | 14 | Axial Pullout | X | | 722.9 |
| 5 | M | 39 | R | 16 | Axial Pullout |  | | 139.8 |
| 6 | F | 39 | L | 13 | Axial Pullout |  | | 244.2 |
| 7 | M | 16 | R | 15 | Axial Pullout |  | | 132.5 |
| 8 | M | 55 | L | 15 | Axial Pullout |  | | 232.9 |
| 9 | M | 19 | R | 15 | Axial Pullout |  | | 134.4 |
|  |  |  |  |  | Average ± STDEV | | 281.7 ± 191.5 | |

| **Specimen #** | **Sex** | **Age** | **Side** | **Size** | **Test** | **Fracture** | | **Ultimate Failure (Nm)** |
| --- | --- | --- | --- | --- | --- | --- | --- | --- |
| 10 | F | 59 | R | 12 | Torsion |  | | 3.7 |
| 11 | F | 87 | L | 16 | Torsion |  | | 2.1 |
| 12 | F | 31 | L | 14 | Torsion | X | | 1.1 |
| 13 | M | 26 | L | 14 | Torsion |  | | 2.5 |
| 14 | F | 42 | L | 13 | Torsion |  | | 4.0 |
| 15 | M | 48 | L | 14 | Torsion |  | | 7.0 |
| 16 | M | 26 | L | 15 | Torsion |  | | 6.5 |
| 17 | M | 35 | L | 16 | Torsion |  | | 6.5 |
|  |  |  |  |  | Average ± STDEV | | 4.2 ± 2.3 | |
